# Treatment of unruptured aneurysms with flow diverting stents is safe and effective

**DOI:** 10.1177/23969873231154062

**Published:** 2023-02-07

**Authors:** Martin Bendszus, Markus A Möhlenbruch

**Affiliations:** Department of Neuroradiology, Heidelberg University Hospital, Heidelberg, Germany

Prof. Dr. Kennedy Lees,

We read with interest the Guidelines of the European Stroke Organisation (ESO) on the management of unruptured intracranial aneurysms (UIA) recently published in The European Stroke Journal.^1^ We congratulate the authors Dr. Etminan et al. for their thorough analysis of risk factors, treatment options, and providing a clear recommendation for the decision to treat UIA, that is, when the estimated treatment risk is lower than the 5-year rupture rate. Moreover, we appreciate the definition of prerequisites of treating UIA, for example, interdisciplinary neurovascular centers with a minimum of caseload per center and physician per year. However, the authors repeatedly state throughout the manuscript that flow diverting stents should only be used when no other treatment option is valid for aneurysm repair. This statement is based on a single small prospective study which was halted after 78 patients due to unexpected morbidity and mortality in the flow diverting stent cohort.^2^ This study goes back to the early days of the application of flow diverting stents and has been criticized for severe methodological flaws.^3^ Briefly, this small exploratory study was performed in a highly selected group of high-risk patients with vague selection criteria, prone to a severe selection bias. Therefore, this study cannot serve as an overall argument against the initial use of flow diverting stents in UIA. In contrast, in the meantime a plethora of retrospective and prospective controlled data is available demonstrating overwhelming safety and efficacy of this treatment option in UIA, not only as last treatment option (recent review and meta-analysis of more than 1000 unruptured aneurysms by Shehata et al.^4^). The statement that flow diverting stents in UIA should only be used as ultimate treatment option is therefore not substantiated by the literature and should be regarded with great caution, as this will put economic and legal pressure on the neurointerventionalist planning and performing the procedure.
